# Mediterranean Diet and Chronic Kidney Disease (CKD): A Practical Approach

**DOI:** 10.3390/nu15010097

**Published:** 2022-12-25

**Authors:** Almudena Pérez-Torres, Alberto Caverni-Muñoz, Elena González García

**Affiliations:** 1 Nutrition Department, Hospital Universitario Santa Cristina, 28009 Madrid, Spain; 2 Nephrology Department, Hospital Universitario La Paz, 28046 Madrid, Spain; 3 Nutrition Department, Hospital Quiron Salud, 50006 Zaragoza, Spain; 4 Nutrition Department, Alcer EBRO, 50006 Zaragoza, Spain

**Keywords:** Mediterranean diet, chronic kidney disease, dietary pattern

## Abstract

Chronic kidney disease has become a serious public health issue, as well as others health problems such as hypertension, DM, and obesity. Mediterranean diet (MD) can reduce the risk of cardiovascular disease and cancer and can lead to weight loss in obesity. There are studies that suggest that MD could be the diet of choice for patients with CKD for its influence on endothelial function, inflammation, lipid profile and blood pressure. There are few studies that tell us how to adapt MD to this group of patients. This review aims to offer a practical approach to Mediterranean diet adaptation as nutritional treatment in CKD patients.

## 1. Introduction

Chronic kidney disease (CKD) is the progressive deterioration of the glomerular filtration rate. Beyond aging, its main frequent causes are hypertension and diabetes mellitus (DM) [1]. Recent studies have shown a relationship between obesity and the worsening of CKD, considering it one of the main factors of de novo CKD [2]. Additionally, high protein intake is related to the development of glomerular hyperfiltration and increased intraglomerular pressure, aggravating the progression of CKD or even producing it [3]. 

Numerous studies suggest that Mediterranean diet (MD) can reduce the risk of cardiovascular disease and cancer and has several possible protectives mechanisms against diabetes mellitus type 2 [4]. A systematic review suggests that MD can be useful for patients with obesity, and its results in weight loss were similar to those of other diets (low-fat diet and low-carbohydrate diet) [5].

A meta-review by Martinez-Lacoba et al aims to summarize, synthesize and organize the effects of MD pattern on different health outcomes. The included studies analyzed the relationship between MD and 14 issues: adherence, all-cause mortality, asthma, cancer, cognitive functioning, cardiovascular disease (CVD), economic evaluation, fractures, health-related quality of life, hypertension, metabolic syndrome, obesity, body weight and body mass index (BMI), rheumatoid arthritis and type 2 diabetes. All of these are catalogued as noncommunicable diseases (NCD). In this meta-review, MD has been shown to be a healthy dietary pattern that may reduce risk related to NCD; the effect is larger if the pattern is combined with physical activity, and tobacco and excessive alcohol consumption are avoided [6].

A recent study conducted in Spanish population determined an inverse relationship between adherence to MD measured by the Mediterranean Diet Adherence Screener (MEDAS-14) and metabolic syndrome in 254 subjects aged between 55 and 80 years [7]. 

A systematic review by Papadaki et al. of 84 papers and 57 controlled trials aimed to evaluate the effect of the MD, compared to usual care, no treatment, or a different diet, on metabolic syndrome incidence, metabolic syndrome, and components and risk factors in addition to related comorbidity outcomes and treatment for these health outcomes. It concluded that the MD beneficially affects several outcomes implicated in metabolic health, including metabolic syndrome components and several metabolic risk factors, in addition to incidence of CVD and stroke [8].

Bash et al, in a systematic review and meta-analysis, concluded that a healthy diet pattern (possibly MD) is characterized by a high consumption of fruits, vegetables, nuts, legumes and whole grains. They also concluded that a low consumption of salt, sugary drinks and meat was associated with a lower incidence of CKD and albuminuria [9]. This same study found no relationship between the healthy eating pattern and the decrease in renal function. However, the Chronic Renal Insufficiency Cohort (CRIC) study, conducted in 2403 patients with eGFR between 20–70 mL/min, concluded that a better adherence to healthy dietary patterns, with similar characteristics to MD, had lower risk of CKD progression and all-cause mortality among people with CKD [10].

The 2020 KDOQI guidelines suggest the Mediterranean diet in adults with CKD 1–5 not on dialysis and in transplant patients to improve lipid control. In CKD 1–4 patients, an increase in fruit and vegetable consumption is recommended in order to reduce body weight, blood pressure and the production of acid load. In the same way, an alkaline diet is recommended in CKD 1–4 patients to reduce the GFR decrease rate [11].

There are studies that suggest the use of MD as the diet of choice for patients with CKD, regardless of the CKD stage, for its benefits for endothelial function, inflammation, lipid profile and blood pressure [12].

The Med Diet 4.0 framework recognizes the MD not only for its major health and nutrition benefits in the prevention of chronic diseases, but also its low environmental impact, richness in biodiversity, reduction of pressure on natural resources and mitigation of climate change [13]. 

This review aims to offer a clinical practical approach to adapt Mediterranean diet as nutritional treatment in CKD patients in their different stages.

## 2. Mediterranean Diet General Description

Although many studies suggest the beneficial effects of MD, there are inconsistencies among the definition and the principal characteristics of MD pattern [14]. The MD, as a traditional dietary pattern, is rich in plant-based foods (cereals, legumes, nuts, fruits, vegetables and herbs) and low in red meat. It includes a moderate intake of fish, seafood, eggs, white meat and dairy products, and a moderate intake of alcohol (mainly wine during meals where is culturally acceptable). Olive oil is the main source of added fat.

Details of the principal characteristics of Mediterranean dietary pattern can be found below [12,15,16]:

- The principal source of fat is extra virgin olive oil. It is added to vegetables and legumes. A meta-analysis of 32 observational studies in general population revealed that extra virgin oil consumption decreased the risk of stroke, CHD (coronary heart disease), and diabetes and improved some metabolic and inflammatory biomarkers [17].
- Noce et al. highlighted the role of extra virgin olive oil in the treatment of CKD and its comorbidity due to its antioxidant and anti-inflammatory extra effects.
- High consumption of vegetable products such as fruits, legumes, mushrooms and whole grains cereals. Fruits are eaten as desserts or snack. In meta-analyses of prospective observational studies, a higher intake of fruit has been associated with lower risk of all-cause mortality, CHD, stroke, type 2 diabetes, CRC, hypertension, and adiposity [18].
- Preferential consumption of seasonal and local foods (sustainable). Consuming a variety of colors in both vegetables and fruits is strongly recommended to help ensure intake of a broad range of micronutrients and phytochemicals.
- Preference is given to plant protein sources such as legumes, but also animal protein sources low in saturated fats. Mediterranean 4.0 framework recommends a daily consumption of legumes in a moderate amount.
- Olives (others than olive oil), nuts and seeds could be consumed daily since they are good sources of unsaturated healthy fats, minerals, vitamins and fiber, as well as antioxidants.
- Consumption of herbs, spices, garlic and onions to increase food palatability and allow a reduction in salt use.
- Lesser consumption of dairy products, and preferent use of yogurt and cheese for their probiotic content, to boost digestive tract health and positively affect the microbiome.
- Two or three times of fish consumption per week (mainly fish rich in omega-3). Eicosapentaenoic (EPA) and docosahexaenoic (DHA) acids are reported to reduce the risk of coronary heart disease and to have anti-inflammatory properties. A systematic review by Schwingshackl L et al. indicated that the risk of mortality decreased by 10% with increasing intake of 200 g fish/day [18].
- Red meats should be eaten less frequently, preferably as lean cuts and in stews with vegetables.
- Whole eggs, including those used for cooking or baking, should not exceed four eggs per week.
- Low to moderate amount of wine consumption in meals. Maximum of one glass per day for women and two glasses per day for men.
- Infrequent consumption of ultra-processed products.
- Portion sizes should be based on frugality and moderation and aligned with the energy needs of urban and modern lifestyles where applicable. Therefore, it is important to provide an indication of the recommended frequency of foods to consume. (Table 1 shows the recommendation of the MD adapted frequency of food).
- An adequate daily hydration (mainly water and non-sweetened beverages). Coffee, tea and herbal infusions (rich in flavonoids) are also included, but they should be consumed preferably without any sweetener.
- A total of 3–5 meals per day.
- Main meals consumed daily should be a combination of three elements: cereals, vegetables and fruits. In addition, a small quantity of legumes, beans or other side dishes should be consumed, though not in every meal.
- Mealtimes have a social and cultural value which transcends their nutritional and nourishing functions.
- Consuming eco-friendly products will help the preservation of Mediterranean landscapes and sea.
- Selection and preference of traditional and local foods will sustain the local culinary heritage.

## 3. Arguments in Favor of Mediterranean Diet (MD) in CKD

The benefits reported in studies in favor of prescribing MD in CKD are [12,19] the following:

- Lower cardiovascular and cancer risk;
- Lower risk of incident CKD;
- Decreased systematic inflammation;
- Improved lipid profile and lipoprotein metabolism;
- Lower blood pressure;
- Beneficial effects on glucose control, hyperinsulinemia, insulin resistance and satiety;
- Reduced oxidative stress;
- Changes in the intestinal microbiota composition. Dietary fiber can change colonic microbial activity from a proteolytic to a saccharolytic fermentation pattern.

The classic guidelines in CKD restricted the consumption of vegetable and whole grain products due to their high levels of potassium and phosphorus. However, current publications recommend increasing their intake because it is not related to serum potassium levels increase. This is due to the fact that the bioavailability of potassium and phosphorus in plant foods is less than 50%, while in the additive of ultra-processed foods it is 100% [20,21].

The benefits of MD can also be transferred to CKD population. The elevated vegetable content present in MD is associated with a low acid load, a reduced progression of CKD, a reduction of constipation and an improvement in the intestinal microbiota due to the increased amount of dietary fiber consumed [12].

The main benefits in the whole-grain product consumption (principal source of insoluble fiber) is a reduction of blood pressure and blood lipid levels and amelioration of constipation [12,22]. 

## 4. Nutritional Requirements According to Stage of Chronic Kidney Disease

The latest KDOQI guidelines [11] recommend an energy intake of 25–35 kcal/kg body weight/day to maintain an adequate nutritional status, considering age, gender, physical activity level, body composition, weight goals, CKD stage, and presence of concurrent disease or inflammation. Ensuring a negative nitrogen balance is necessary to maintain protein deposits and reduce the high risk of malnutrition in this group of patients. Therefore, in hypercatabolic patients, regardless of the degree of CKD, an energy intake of 30–35 kcal/kg/day is recommended [23]. In addition, the KDOQI guidelines do not recommend phosphorus and potassium intake restriction, unless the biochemical blood values are altered. There is no recommendation about fiber or polyunsaturated fatty acids (PUFA), which are important elements in MD.

Protein intake recommendations are dynamic and vary depending on the CKD stage (Table 2). There is no recommendation about the origin of the protein, animal or vegetable. 

## 5. Mediterranean Diet Adaptation at Different Stages of Chronic Kidney Disease

Nutrition is a fundamental pillar in CKD treatment, with differences in requirements according to CKD stage. Following the recommendations of the K/DOQI guidelines [9], we have adapted the Mediterranean diet recommendations to the different stages of CKD. Table 3 shows the frequency of recommendation foods adapted to MD and CKD.

- Protein intake is adjusted depending on the stage of CKD, so that is important to adapt the serving size to reach the recommendation in each stage.
- Principal nutritional problems in CKD are related to potassium, phosphorus, and sodium intake. Potassium and phosphate additives are abundant in food supply and are highly bioavailable, so that the principal CKD diet recommendation must be to limit ultra-processed products despite the actual recommendation indicated to only restrict it if necessary.

There are cooking methods to decrease the potassium levels in vegetable food [24]. It is recommended to consume canned or frozen vegetables and legumes applying the following cooking techniques:

- Vegetable or legume preserved/canned: Remove the liquid from the preserve, wash it well under the tap and apply a short cooking time of 4–5 min.
- Frozen vegetables or legumes: Thaw them in water for 2–3 h, then remove the soaking water and cook them in clean water for as long as necessary.
- To consume fresh vegetables or vegetables cooked without water (grill, oven, etc.): peel them (if possible), chop them and extend the soaking time to 8 h with two changes of water.
- To consume dried legumes: soak for 12 h with two changes of water, discard the soaking water and apply the necessary cooking time.

In relation to phosphorus level, if it is elevated, it is advisable to use the phosphorus/protein ratio (ratio between organic phosphorus and protein content in food). It is recommended to consume a phosphorus/protein food ratio of lower than 12 md/g, and to limit the intake of food with a ratio phosphorus/protein of higher than 16 mg/day [25]. 

The principal recommendations to sodium intake are the following:

- Reduce the use of salt;
- Limit intake of smoked, salted, and processed meats;
- Avoid canned foods, cured cheeses, shellfish, and crustaceans;
- Lessen the consumption of vegetable pickles and commercial sauces.

### 5.1. CKD without Dialysis

The KDOQI guidelines [11] recommend a low-protein diet that provides 0.55–0.6 g/kg/day for patients with CKD stages 3–5 not on dialysis who are metabolically stable, or a very low–protein diet that provides 0.28–0.43 g/kg/day plus the use of ketoanalogues. For the earlier stages (1–3) of the disease, no clear recommendation exists, although some guidelines suggest the use of moderately low-protein diets of 0.8–1 g protein/kg bodyweight/day [23,24]. 

To reach the protein requirements without decreasing the energy intake, it is necessary to take into account the serving of protein foods, especially animal products (meat, fish, eggs and dairy products). The best option is vegetable protein (legumes).

If potassium levels are high, our nutritional recommendations, based on our clinical experience and scientific evidence, are the following:

- First step: look for other possible causes, apart from intake, such as drugs, constipation, prolonged fasting or poor metabolic glucose control [26].
- Second step: Reduce the intake of ultra-processed foods due to their high content of inorganic potassium [27].
- Third step: Consider the amount of potassium per portion of food served, and the potassium/fiber ratio. There are currently no validated reference values for these tools in patients with CKD [28].
- Fourth step: Educate yourself in the recommended culinary techniques to reduce potassium in food.

Finally, restrict foods with a high potassium content, and that cannot be subjected to culinary techniques to reduce it. For example, chocolate.

In the same way, if phosphorus values are high, we recommend taking the following steps:

1. Reduce ultra-processed foods, as the main source of additives, and inorganic phosphorus.
2. Educate yourself on the selection of foods with a P/protein ratio of < 16mg/g, and if the values remain high, consume foods with a P/protein ratio of < 12mg [25].

In relation to reducing sodium intake, we recommend the following:

1. Reduce ultra-processed foods, as they are the main source of dietary sodium.
2. Moderate the consumption of common salt while retaining tools to flavor the dishes such as spices. Consider the number of spices to use according to the little information available on patients with CKD.

### 5.2. Hemodialysis

Protein Energy Wasting (PEW) increases the risk of mortality from various causes. This has been demonstrated in all phases of CKD [29], but it is decisive in patients on hemodialysis and those starting dialysis techniques [30,31,32]. Current guidelines recommend that metabolically stable adults with CKD stage 5 on hemodialysis or peritoneal dialysis have a protein intake of 1–1.2 g/kg/day to maintain an adequate nutritional status. In adults with DM (at risk of hyperglycemia or hypoglycemia), higher protein levels may be considered to achieve glycemic control [11].

In hemodialysis, is recommended to limit the liquid intake. Be aware of herb drink-stimulated diuresis. In order to reduce fluid intake, the following is recommended:

- Use preferably water;
- Avoid the use of sugary drinks, and limit salt intake, because they increase the sensation of thirst;
- There is no information regarding the consumption of infusions in any of the stages of CKD.

If potassium or phosphorus values are high, or it is necessary to restrict sodium levels, we refer to the recommendations provided in Section 5.1.

### 5.3. Peritoneal Dialysis

Current protein intake recommendations are the same in hemodialysis as in peritoneal dialysis (1 to 1.2 g/kg of weight/day). However, various guidelines recommend a protein intake higher than 1.2 g/kg/day [29,30,31], increasing to 1.5 g/kg/day in case of peritonitis [33]. 

One of the main factors determining the nutritional status and dietary recommendations of these patients is the presence of glucose in dialysis solutions and its absorption via the peritoneal route [34,35].

To minimize protein catabolism, an energy intake of 25–35 kcal/kg/day is recommended, considering the amount of glucose absorbed via the peritoneal route, by either direct quantification or the use of standard formulas [36]. Therefore, it is important to limit the carbohydrate intake in the form of products with added content sugar, and if necessary, to decrease the cereal serving/day.

The current literature on the nutritional treatment of PD patients is limited. A Mexican group indicates the importance of considering the type of membrane transporter in these patients. In high transporters patients, they recommend a protein intake of > 1.2g/kg weight/day, always including vegetables to maintain the acid load of the diet as well as a greater limitation of carbohydrates (45–55%), considering that its absorption via the peritoneal route is greater [37].

Constipation is common in PD patients, so that the whole grain, legumes and vegetable intake can contribute to ameliorate it.

If potassium or phosphorus values are high, or it is necessary to restrict sodium levels, we refer to the recommendations given in Section 5.1.

### 5.4. Transplant

No evidence exists for protein recommendations in kidney transplantation. The KDOQI guidelines [15] offer no specific recommendations. A subsequent consensus document recommends a moderate protein intake of 0.8–1 g/kg/day [38]. In case of proteinuria or a GFR of <45 mL/min/1.73 m^2^, a 0.6–0.8 g/kg/day intake is recommendable, with at least 50% of the high biological value proteins [30]. However, this is not specific to transplant patients.

After kidney transplantation, the number of dietary restrictions decreases. Some studies have reported a change in eating patterns and an increased fat consumption, but we have discovered no study of dietary intervention showing effects in this area [39,40]

The KDOQI guidelines [15] recommend the MD pattern in these patients without adaptations.

## 6. Conclusions

CKD is one of the most prevalent pathologies frequently arising due to factors that have become a serious public health issue, such as hypertension, DM, and obesity. The MD pattern is associated with a reduction in blood pressure, and the risk of DM, obesity or CVD disease, so it could have positive effects on CKD. In addition, the MD has demonstrated benefits in CKD, such as a reduced the acid load diet, improved microbiota, a decreased inflammation and amelioration of constipation. 

A higher intake of vegetables, fruits and whole grains, as well as a moderate consumption of animal foods contribute to decrease the phosphorus and potassium blood levels. A reduction of ultra-processed product intake is associated with lower levels of sodium, potassium, and phosphorus. MD diet pattern is recommended to CKD patients with adaptations depending on the CKD stage. 

To summarize:

1. The DM can be adapted to any stage of CKD.
2. Food restriction should only be used in the case in which the analytical values are altered.
3. It is advisable at any stage of CKD to limit the consumption of ultra-processed foods due to their content of potassium, phosphorus and sodium since its bioavailability is 100% compared to organic sources.
4. Increasing the consumption of fruits, vegetables and legumes in patients with CKD has several benefits, although it has not been shown that they are responsible for the increase in serum potassium levels.
5. We consider its individualization as a key element of nutritional treatment.

## Figures and Tables

**Table 1:** Recommendations of the frequency of food adapted to Mediterranean diet (MD) [12,14].

Food	Portion Size (Raw)	Serving Size/Day
Cereals (bread, pasta, rice, potatoes and others) Preferably whole grains	Bread: 30–60 g Rice, polenta, couscous or pasta: 50–80 g Potatoes: 150–200 g	5–6 Adapted to physical activity
Vegetables	150–250 g	Minimum 2
Fruits	150–200 g	Minimum 3
Dairy products Preferably low-fat products, and sugar-free	Milk: 200–250 mL 2 Yoghurt: 125 mL Low-fat cheese: 85–125 g Normal cheese: 40–60 g	Maximum 3
Extra virgin olive oil	10 mL	4–6 Adapted to energy requirements
Legumes	50–60 g	Minimum 4 servings/week
Fish Preferably omega-3 fish	125–150 g	Minimum 3 servings/week
Eggs	53–63 g	Maximum 4 servings/week
Meat Preferably lean meat: poultry products	100–125 g	Maximum 3 servings/week
Nuts, seeds and olives. Preferably without salt, sugar and fat	20–30 g	Minimum 3 servings/week. Preferably daily

**Table 2:** Nutritional and protein requirements according to chronic kidney disease stage [11,23].

	Energy (kcal/kg/day) ^a^	Protein (g/kg/day)	% Lipids/HC of Total TCV	Potassium (mg/day) ^d^	Phosphorus (mg/day) ^d^	Sodium (g/day)
Stages 1–2	30–35	0.8 + proteinuria ^c^	(30–35/50–60)	Individualize	Individualize	2.3
Stages 3–5, no Dialysis	30–35	0.55–0.6 + proteinuria ^c^ DM: 0.6–0.8	(30–35/50–60)	Individualize If it is elevated: 1500–2000	Individualize If it is elevated: 600–1000	2.3
Hemodialysis	30–35	1–1.2	(30–35/45–55)	Individualize If it is elevated: 1500–2000	Individualize If it is elevated: 800–1000	2.3
Peritoneal Dialysis	30–35 ^b^	1–1.2	(30–35/45–55)^b^	Individualize If it is elevated: 1500–2000	Individualize If it is elevated: 600–1000	2.3
Transplant	30–35	1 + proteinuria ^c^	30–35/50–60	Individualize	Individualize	2.3

**Notes:**^a^ In case of obesity (BMI ≥ 30 kg/m^2^), calculate requirements according to adjusted weight or BMI = 23 kg/m^2^. ^b^ Count glucose absorption. ^c^ In case of proteinuria, increase protein intake by 1 g of protein per g of proteinuria in 24-h urine volume. ^d^ Individualize according to analytical values. DM: Diabetes mellitus, TCV: total caloric value.

**Table 3:** Frequency recommendation foods adapted to Mediterranean diet (MD) and chronic kidney disease (CKD) stage [11,12].

Food Groups	Stage 1–2	Stages 3–5, no Dialysis	Hemodialysis	Peritoneal Dialysis	Transplant
Cereals (bread, pasta, rice, potatoes and others). Preferably whole grains	5	6	5	4	5
Vegetables	2	2	2	2	2
Fruits	3	2	2	2–3	3
Dairy products Preferably low-fat sugar-free products	1.5	1.5	1	1	1.5
Extra virgin olive oil	6	6	6	7	6
Legumes	Minimum 4 servings/week	Minimum 4 serving/week	2–3 servings/week	2–3 servings/week	Minimum 4 servings/week
Fish Preferably omega-3 fish. Adjusted ratio P/proteins	Minimum 3 servings/week	Minimum 2 serving/week Adjusted ratio P/proteins	Minimum 3 servings/week Adjusted ratio P/proteins	Minimum 3 servings/week Adjusted ratio P/proteins	Minimum 3 servings/week
Eggs	Maximum 4 servings/week	Maximum 3 serving/week	Maximum 3 servings/week	Maximum 4 servings/week	Maximun 4 servings/week
Meat Preferably lean meat: poultry products	Maximum 3 servings/week	Maximum 2 serving/week Adjusted ratio P/proteins	Maximum 3 servings/week Adjusted ratio P/proteins	Maximum 3 servings/week Adjusted ratio P/proteins	Maximum 3 servings/week
Nuts or seeds Preferably without salt, sugar or fat	1	Individualize	Individualize	Individualize	1
